# Functional near‐infrared spectroscopy study of the neural correlates between auditory environments and intellectual work performance

**DOI:** 10.1002/brb3.1104

**Published:** 2018-09-05

**Authors:** Satoru Hiwa, Tomoka Katayama, Tomoyuki Hiroyasu

**Affiliations:** ^1^ Doshisha University Kyotanabe‐Shi Kyoto Japan

**Keywords:** auditory environment, functional connectivity, functional near‐infrared spectroscopy (fNIRS), graph theoretical analysis, intellectual work performance, number memory task

## Abstract

**Introduction:**

Many people spend a considerable amount of time performing intellectual activities within auditory environments that affect work efficiency. To investigate auditory environments that improve working efficiency, we investigated the relationship between brain activity and performance of the number memory task in environments with and without white noise using functional near‐infrared spectroscopy (fNIRS).

**Methods:**

Twenty‐nine healthy subjects (aged 21.9 ± 1.4 years) performed the number memory task in both the white noise and silent environments. Cerebral blood flow changes during the task were measured using an ETG‐7100 fNIRS system (Hitachi, Ltd., Tokyo, Japan). The psychological states of the subjects were also estimated by subjective ratings of the pleasantness of the auditory environment. Then, they were divided into three groups based on their task scores. The differences in the cerebral blood flow (CBF) changes, functional connection strength, and the subjects’ feelings of pleasantness to the noise between the subject groups were analyzed and discussed.

**Results:**

The first group felt that the white noise was pleasant, which strengthened the bilateral functional connections between the brain regions related to the memory task. Therefore, the subjects’ task performance improved in the white noise environment. Although the second group felt that the white noise was uncomfortable, the frontal regions related to attention control were more activated in the white noise environment to sustain the task performance in the noisy environment. The third group felt that the white noise was unpleasant, and their CBF decreased in that environment, which was associated with deteriorated task performance.

**Conclusions:**

Task performance was closely related to the subjects’ feelings of pleasantness to the noise. The results of the analysis of the CBF changes and functional connectivity suggested that the effects of the white noise on brain activity differed among the three groups.

## 1 INTRODUCTION

In modern society, people spend a considerable amount of time on intellectual activities, such as office work and learning. They may feel fatigued and stressed due to external environmental factors, such as light levels, sound levels, and temperature, which has resulted in the concern that work efficiency could deteriorate consequently (Chellappa et al., 2011; Hygge & Knez, 2001; Kwon, Chun, & Kwak, 2011; Lamb & Kwok, 2016). Optimal environmental conditions enable people to concentrate on their intellectual work to improve their performance efficiency. Previous studies have revealed that music affects mood and arousal (Husain, Thompson, & Schellenberg, 2002; Panksepp & Bernatzky, 2002). Furthermore, multiple studies have investigated the influence of sound, such as music, in the environment. For example, Thompson et al. have reported that Mozart’s music positively affects arousal state and mood, which improves performances involving spatial abilities (Thompson, Schellenberg, & Husain, 2001). Moreover, Cassidy et al. have revealed that the effects of music on the performance of cognitive tasks differ according to people’s personalities (Cassidy & MacDonald, 2007). Perham et al. have reported that recall task performance declines regardless of music preference (Perham & Vizard, 2011). The results of these studies indicate that the effects of sound on the efficiency of intellectual work performance vary according to the sound and personality of the subject. In particular, the efficiency of intellectual work is closely related to brain activity.

However, although many studies have evaluated subject states using task performance or questionnaires, the brain function mechanisms associated with the effects of sound on work efficiency are not clear. Because the brain plays a central role in activities, such as thought and emotion, evaluations of the effects of sound environments on work efficiency should be possible by analyzing brain activities. Therefore, the purpose of this study was to quantify the effects of sound using brain activities.

The effects of the sound environment on physiological indicators and brain functions have been investigated by multiple studies (Blood & Zatorre, 2001; Bodner, Muftuler, Nalcioglu, & Shaw, 2001; Rauscher et al., 1995; Sarnthein et al., 1997). For example, electroencephalographic investigations have revealed that Mozart’s music enhances learning by activating the task‐related brain regions (Jaušovec et al., 2006). In addition, positron emission tomography scans and electroencephalography have shown that cognitive processes and music interact (Nakamura et al., 1999). Although the effects of music on brain activity have been studied as described above, the effects of environmental sound on intellectual work that involves brain activity have not been sufficiently investigated.

Functional near‐infrared spectroscopy (fNIRS) monitors cerebral blood flow [CBF; hemoglobin (Hb) concentration] and shows the hemodynamic responses of oxy‐ and deoxy‐Hb. This technique has therefore been widely used not only for research on brain functions and cognitive activities but also for the diagnosis and treatment of mental illnesses (Ferrari & Quaresima, 2012; Hoshi et al., 2003). Because the operating noise and size of fNIRS devices are less than those of functional magnetic resonance imaging scanners, we can measure brain activity during daily work performance in the natural state (Plichta et al., 2011). Because of these advantages, we used fNIRS to investigate the effects of environmental sound on intellectual work.

Previous studies have revealed that acoustic noise has detrimental or beneficial effects on the performance of intellectual work (Dalton & Behm, 2007; Kujala & Brattico, 2009; Szalma & Hancock, 2011). In addition, white noise has been reported to enhance cognitive performance, with the amount of effect on performance differing according to attention level (Söderlund, Sikström, & Smart, 2007; Söderlund, Sikström, Loftesnes, & Sonuga‐Barke, 2010). However, these findings have not been verified using physiological indicators. Therefore, in this study, the relationship between the performance of intellectual work and CBF changes was investigated in white noise and silent environments. In particular, because we assumed that office workers performed short‐term memory tasks and computer data entry in their daily work (Jaušovec & Habe, 2005; Miller, 1956), a number memory task was used as the intellectual work in this study. The relationship between brain activity and the psychological state of the subject that was estimated using questionnaires was also examined.

## 2 MATERIALS AND METHODS

### 2.1 Participants

Twenty‐nine healthy subjects (age, 21.9 ± 1.4 years; 14 females, 27 right‐handed) participated in this experiment after providing written informed consent. The experiments were conducted from 10:00 a.m. to 5:00 p.m. (17:00), and the room temperature and humidity were controlled during the experiment (23.7 ± 22.7°C and 64.0% ± 6.2%, respectively). The participants performed intellectual work in two auditory environments, and their brain activity during the task was measured using fNIRS. After the tasks and measurements were conducted, the subjects were asked to rate their feelings of pleasantness to the auditory stimulus presented in the experiment.

### 2.2 Auditory environments

In this experiment, the participants performed the number memory task in both the white noise and silent environments. White noise was presented through speakers (MSS50 multimedia speaker system; SOTEC Co., Ltd., Yokohama, Japan) positioned on the left and right sides of a liquid crystal display (LCD) monitor. The sound level was set to 65 ± 1 dB, and both the white noise and silent environments also included the sounds associated with operating the fNIRS device (47 ± 1 dB). The noise level of the typical office environment is about 60 dB, while that of a quiet place, such as a library, is about 40 dB [Federal Interagency Committee on Noise (US) (1992)]. The experiments were conducted in both environments on the same day, and the order of the presentation of the environmental conditions was randomized for each participant.

### 2.3 Behavioral data acquisition

#### 2.3.1 Number memory task

**Figure 1:**
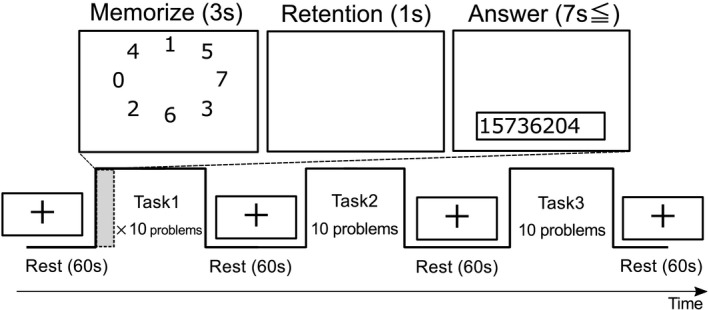
Experimental design. The resting block, which consisted of 60 s of gazing at the fixation point “+” in the center of the screen, was followed by the number memory task block. Each task block included 10 trials of the number memory task, and each trial was composed of the following three steps: memorize, retain, and answer. During the memorization step, the subjects memorized the eight single‐digit numbers that were displayed in a circle for 3 s on the monitor. The participants were then required to retain the memorized numbers during the 1‐s retention step. Finally, in the answer step, they entered the remembered numbers in clockwise order within 7 s. This procedure was repeated three times. The total duration of the experiment differed for each participant because the time required for giving answers differed among them

A number memory task that required short‐term memory and computer data entry were used to simulate intellectual work in a real office environment. The participants were asked to memorize eight single‐digit numbers displayed on the LCD monitor. The design of the task is illustrated in Figure 1. The resting block, which consisted of 60 s of gazing at the fixation point “+” in the center of the screen, was followed by the number memory task block. The participants moved their fingers minimally during the resting block. During the memorization step, the participants memorized the eight single‐digit numbers that were displayed in a circle on the LCD monitor for 3 s. The number of characters presented was set to eight because reports have stated that we can memorize only six to eight characters in a few seconds. The participants were then required to retain the memorized numbers for 1 s in the retention step. Finally, during the answer step, the participants entered the remembered numbers in clockwise order within 7 s. The duration of the answer block varied depending on the subject because the next problem was shown as soon as they were able to answer the current problem within 7 s. The task block followed the resting block, and these blocks were repeated three times. An extra resting block was provided following the third task block. Therefore, the total duration of the experiment also differed in each participant. This procedure was performed separately in the two environmental conditions. The percentage of correct answers was used as the performance measure of the number memory task.

#### 2.3.2 Subjects’ ratings of feelings of pleasantness

After finishing the tasks and measurements, the participants were asked to rate their level of pleasant feelings to the auditory environment on a visual analog scale (VAS) (Bond & Lader, 1974; Maxwell, 1978). The VAS rating was a continuum along a solid line that ranged from 0 (unpleasant) to 10 (pleasant). The VAS was scored as a continuous value according to the distance from one of the two anchors of the rating line. Because we recorded the subjective rating as a continuous value, the VAS scores were compared between the two environments using a *t* test (Dexter & Chestnut, 1995; Philip, 1990).

### 2.4 fNIRS data acquisition

**Figure 2:**
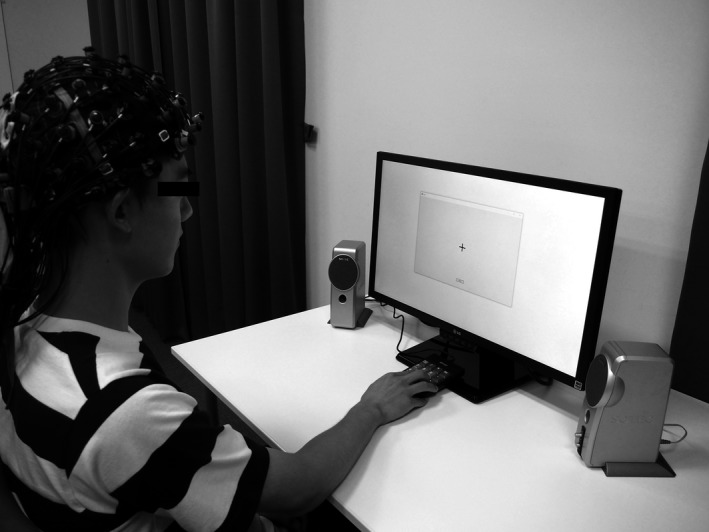
Experimental environment. The participants performed the number memory task in front of the monitor and responded using the numeric keypad. The distance between the monitor and the subject was adjusted to 50 cm

CBF changes were measured using an ETG‐7100 fNIRS system (Hitachi, Ltd., Tokyo, Japan) with a sampling frequency of 10 Hz. The fNIRS probes were placed according to the International 10–20 system, and the frontal (22CH) and left/right temporal (24CH for each) regions were measured. As shown in Figure 2, the participants performed the number memory task in front of the LCD monitor and responded using the numeric keypad. The distance between the monitor and the subject was adjusted to 50 cm.

### 2.5 Data preprocessing

**Figure 3:**
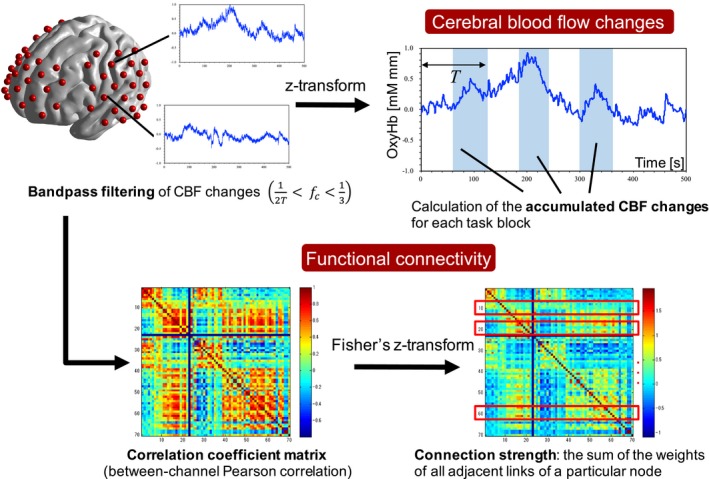
Computational flowchart of the analysis of functional near‐infrared spectroscopy (fNIRS) time series. The fNIRS data were band‐pass‐filtered with a passband (fc), which was the inverse number of twice each trial period (T) Hz to 0.33 Hz. The cerebral blood flow (CBF) change data of each participant were converted to Z‐scores for each measurement channel. The Z‐transformed data were baseline‐corrected so that the value at the beginning of the task block became zero, and the cumulative changes during the task block were calculated. The Pearson correlations among the 70 channels of the oxygenated hemoglobin (oxy‐Hb) time course during the task block were calculated for each auditory environmental condition. Functional connection strength, which was one of the node centrality metrics in graph theory, was calculated for each correlation matrix

Figure 3 shows the procedures involved in the fNIRS data processing. The fNIRS data were band‐pass‐filtered with a passband that was the inverse number of twice each trial period Hz to 0.33 Hz. The measurement channels in which the CBF changes were consistently zero during the measurements were excluded from the analysis because they were considered nondetectable channels. Furthermore, the oxy‐Hb changes per unit time that exceeded 0.1 mMmm were considered motion artifacts (Pena et al., 2003), and the task blocks that contained these changes were also excluded from the analysis. Moreover, spatial registration of the fNIRS channel location to the Automated Anatomic Labeling atlas in Montreal Neurological Institute space was performed using probabilistic registration and virtual registration toolboxes (available at https://www.jichi.ac.jp/brainlab/tools.html) on a platform for optical topography analysis tools (POTATo) that was developed by Hitachi, Ltd. Tables 1, 2, 3 indicate the brain regions that were the estimated locations of the fNIRS channels for the White, Average, and Silence groups, respectively.

**Table 1:** Spatial registration of the fNIRS channel location to the AAL atlas in MNI space for White group

CH number	Region
1	21—Middle temporal gyrus: 100.00%
2	21—Middle temporal gyrus: 64.71%
3	37—Fusiform gyrus: 59.39%
4	48—Retrosubicular area: 44.02%
5	21—Middle temporal gyrus: 57.62%
6	21—Middle temporal gyrus: 44.67%
7	37—Fusiform gyrus: 76.23%
8	43—Subcentral area: 53.50%
9	22—Superior temporal gyrus: 73.38%
10	37—Fusiform gyrus: 39.00%
11	6—Premotor and supplementary motor cortex: 50.87%
12	2—Primary somatosensory cortex: 38.60%
13	22—Superior temporal gyrus: 50.64%
14	39—Angular gyrus part of Wernicke’s area: 34.66%
15	6—Premotor and supplementary motor cortex: 34.58%
16	40—Supramarginal gyrus part of Wernicke’s area: 40.89%
17	40—Supramarginal gyrus part of Wernicke’s area: 38.82%
18	6—Premotor and supplementary motor cortex: 68.82%
19	1—Primary somatosensory cortex: 36.67%
20	40—Supramarginal gyrus part of Wernicke’s area: 90.44%
21	39—Angular gyrus part of Wernicke’s area: 71.37%
22	6—Premotor and supplementary motor cortex: 63.07%
23	3—Primary somatosensory cortex: 29.73%
24	40—Supramarginal gyrus part of Wernicke’s area: 52.66%
25	20—Inferior temporal gyrus: 55.92%
26	21—Middle temporal gyrus: 56.38%
27	21—Middle temporal gyrus: 100.00%
28	37—Fusiform gyrus: 86.62%
29	21—Middle temporal gyrus: 52.41%
30	21—Middle temporal gyrus: 70.29%
31	48—Retrosubicular area: 49.41%
32	37—Fusiform gyrus: 38.22%
33	22—Superior temporal gyrus: 71.51%
34	43—Subcentral area: 44.68%
35	37—Fusiform gyrus: 47.99%
36	22—Superior temporal gyrus: 60.14%
37	43—Subcentral area: 52.51%
38	6—Premotor and supplementary motor cortex: 51.68%
39	40—Supramarginal gyrus part of Wernicke’s area: 34.03%
40	2—Primary somatosensory cortex: 54.93%
41	6—Premotor and supplementary motor cortex: 38.32%
42	39—Angular gyrus part of Wernicke’s area: 90.81%
43	40—Supramarginal gyrus part of Wernicke’s area: 93.47%
44	3—Primary somatosensory cortex: 36.82%
45	6—Premotor and supplementary motor cortex: 61.93%
46	40—Supramarginal gyrus part of Wernicke’s area: 63.27%
47	3—Primary somatosensory cortex: 35.41%
48	6—Premotor and supplementary motor cortex: 73.74%
49	10—Frontopolar area: 58.57%
50	10—Frontopolar area: 86.44%
51	10—Frontopolar area: 69.98%
52	46—Dorsolateral prefrontal cortex: 55.08%
53	46—Dorsolateral prefrontal cortex: 79.07%
54	10—Frontopolar area: 93.48%
55	10—Frontopolar area: 100.00%
56	10—Frontopolar area: 67.15%
57	45—pars triangularis Broca’s area: 52.63%
58	46—Dorsolateral prefrontal cortex: 83.37%
59	10—Frontopolar area: 63.43%
60	10—Frontopolar area: 49.58%
61	46—Dorsolateral prefrontal cortex: 73.30%
62	45—pars triangularis Broca’s area: 34.20%
63	9—Dorsolateral prefrontal cortex: 88.24%
64	9—Dorsolateral prefrontal cortex: 87.02%
65	9—Dorsolateral prefrontal cortex: 64.57%
66	45—pars triangularis Broca’s area: 51.54%
67	9—Dorsolateral prefrontal cortex: 73.49%
68	9—Dorsolateral prefrontal cortex: 65.34%
69	9—Dorsolateral prefrontal cortex: 75.80%
70	9—Dorsolateral prefrontal cortex: 69.88%

Note

fNIRS: functional near‐infrared spectroscopy.

**Table 2:** Spatial registration of the fNIRS channel location to the AAL atlas in MNI space for Average group

CH number	Region
1	21— Middle temporal gyrus: 100.00%
2	21—Middle temporal gyrus: 51.22%
3	37—Fusiform gyrus: 67.43%
4	48—Retrosubicular area: 52.17%
5	21—Middle temporal gyrus: 75.77%
6	21—Middle temporal gyrus: 44.35%
7	37—Fusiform gyrus: 76.97%
8	43—Subcentral area: 41.39%
9	22—Superior temporal gyrus: 71.33%
10	37—Fusiform gyrus: 56.27%
11	6—Premotor and supplementary motor cortex: 71.65%
12	43—Subcentral area: 45.88%
13	22—Superior temporal gyrus: 65.67%
14	37—Fusiform gyrus: 44.92%
15	43—Subcentral area: 38.89%
16	2—Primary somatosensory cortex: 45.13%
17	39—Angular gyrus part of Wernicke’s area: 35.12%
18	6—Premotor and supplementary motor cortex: 63.53%
19	1—Primary somatosensory cortex: 41.80%
20	40—Supramarginal gyrus part of Wernicke’s area: 99.03%
21	39—Angular gyrus part of Wernicke’s area: 88.31%
22	6—Premotor and supplementary motor cortex: 72.12%
23	1—Primary somatosensory cortex: 34.70%
24	40—Supramarginal gyrus part of Wernicke’s area: 56.65%
25	37—Fusiform gyrus: 55.38%
26	20—Inferior temporal gyrus: 53.94%
27	21—Middle temporal gyrus: 92.39%
28	37—Fusiform gyrus: 73.22%
29	20—Inferior temporal gyrus: 43.51%
30	21—Middle temporal gyrus: 79.24%
31	48—Retrosubicular area: 37.43%
32	37—Fusiform gyrus: 54.31%
33	22—Superior temporal gyrus: 59.70%
34	48—Retrosubicular area: 32.33%
35	37—Fusiform gyrus: 48.00%
36	22—Superior temporal gyrus: 66.06%
37	43—Subcentral area: 42.03%
38	6—Premotor and supplementary motor cortex: 47.02%
39	39—Angular gyrus part of Wernicke’s area: 31.96%
40	2—Primary somatosensory cortex: 53.54%
41	43—Subcentral area: 34.46%
42	39—Angular gyrus part of Wernicke’s area: 92.37%
43	40—Supramarginal gyrus part of Wernicke’s area: 91.28%
44	3—Primary somatosensory cortex: 35.89%
45	6—Premotor and supplementary motor cortex: 60.48%
46	40—Supramarginal gyrus part of Wernicke’s area: 49.49%
47	40—Supramarginal gyrus part of Wernicke’s area: 31.53%
48	6—Premotor and supplementary motor cortex: 77.27%
49	10—Frontopolar area: 38.11%
50	10—Frontopolar area: 66.27%
51	11—Orbitofrontal area: 47.76%
52	46—Dorsolateral prefrontal cortex: 50.79%
53	46—Dorsolateral prefrontal cortex: 68.29%
54	10—Frontopolar area: 84.28%
55	10—Frontopolar area: 100.00%
56	10—Frontopolar area: 78.15%
57	46—Dorsolateral prefrontal cortex: 54.73%
58	46—Dorsolateral prefrontal cortex: 78.90%
59	10—Frontopolar area: 81.39%
60	10—Frontopolar area: 71.98%
61	46—Dorsolateral prefrontal cortex: 75.66%
62	45—pars triangularis Broca’s area: 52.72%
63	9—Dorsolateral prefrontal cortex: 64.27%
64	9—Dorsolateral prefrontal cortex: 68.93%
65	9—Dorsolateral prefrontal cortex: 54.39%
66	45—pars triangularis Broca’s area: 69.66%
67	9—Dorsolateral prefrontal cortex: 83.33%
68	9—Dorsolateral prefrontal cortex: 85.37%
69	9—Dorsolateral prefrontal cortex: 92.34%
70	9—Dorsolateral prefrontal cortex: 76.67%

Note

fNIRS: functional near‐infrared spectroscopy.

**Table 3:** Spatial registration of the fNIRS channel location to the AAL atlas in MNI space for Silence group

CH number	Region
1	21—Middle temporal gyrus: 100.00%
2	20—Inferior temporal gyrus: 64.13%
3	37—Fusiform gyrus: 75.99%
4	48—Retrosubicular area: 52.96%
5	21—Middle temporal gyrus: 77.64%
6	20—Inferior temporal gyrus: 41.53%
7	37—Fusiform gyrus: 74.41%
8	43—Subcentral area: 38.10%
9	22—Superior temporal gyrus: 69.63%
10	37—Fusiform gyrus: 57.63%
11	6—Premotor and supplementary motor cortex: 53.79%
12	43—Subcentral area: 41.53%
13	22—Superior temporal gyrus: 60.56%
14	37—Fusiform gyrus: 46.90%
15	6—Premotor and supplementary motor cortex: 41.73%
16	40—Supramarginal gyrus part of Wernicke’s area: 41.06%
17	39—Angular gyrus part of Wernicke’s area: 35.06%
18	6—Premotor and supplementary motor cortex: 58.10%
19	1—Primary somatosensory cortex: 33.77%
20	40—Supramarginal gyrus part of Wernicke’s area: 85.84%
21	39—Angular gyrus part of Wernicke’s area: 85.81%
22	6—Premotor and supplementary motor cortex: 69.99%
23	3—Primary somatosensory cortex: 27.51%
24	40—Supramarginal gyrus part of Wernicke’s area: 53.97%
25	37—Fusiform gyrus: 60.20%
26	20—Inferior temporal gyrus: 74.03%
27	21—Middle temporal gyrus: 100.00%
28	37—Fusiform gyrus: 86.50%
29	20—Inferior temporal gyrus: 59.84%
30	21—Middle temporal gyrus: 85.23%
31	48—Retrosubicular area: 50.60%
32	37—Fusiform gyrus: 54.70%
33	22—Superior temporal gyrus: 62.70%
34	48—Retrosubicular area: 37.04%
35	37—Fusiform gyrus: 54.66%
36	22—Superior temporal gyrus: 70.40%
37	43—Subcentral area: 54.95%
38	44—pars opercularis part of Broca’s area: 54.77%
39	22—Superior temporal gyrus: 31.82%
40	2—Primary somatosensory cortex: 58.17%
41	6—Premotor and supplementary motor cortex: 44.97%
42	39—Angular gyrus part of Wernicke’s area: 86.18%
43	40—Supramarginal gyrus part of Wernicke’s area: 92.18%
44	3—Primary somatosensory cortex: 36.57%
45	9—Dorsolateral prefrontal cortex: 46.15%
46	40—Supramarginal gyrus part of Wernicke’s area: 63.65%
47	3—Primary somatosensory cortex: 37.31%
48	6—Premotor and supplementary motor cortex: 86.86%
49	10—Frontopolar area: 40.46%
50	10—Frontopolar area: 56.98%
51	10—Frontopolar area: 53.90%
52	46—Dorsolateral prefrontal cortex: 48.84%
53	46—Dorsolateral prefrontal cortex: 64.32%
54	10—Frontopolar area: 82.16%
55	10—Frontopolar area: 100.00%
56	10—Frontopolar area: 58.96%
57	45—pars triangularis Broca’s area: 60.12%
58	46—Dorsolateral prefrontal cortex: 93.75%
59	10—Frontopolar area: 86.26%
60	10—Frontopolar area: 61.04%
61	46—Dorsolateral prefrontal cortex: 64.66%
62	45—pars triangularis Broca’s area: 52.48%
63	9—Dorsolateral prefrontal cortex: 77.96%
64	9—Dorsolateral prefrontal cortex: 82.90%
65	9—Dorsolateral prefrontal cortex: 57.12%
66	45—pars triangularis Broca’s area: 56.37%
67	9—Dorsolateral prefrontal cortex: 92.39%
68	9—Dorsolateral prefrontal cortex: 90.94%
69	9—Dorsolateral prefrontal cortex: 90.21%
70	9—Dorsolateral prefrontal cortex: 69.97%

Note

fNIRS: functional near‐infrared spectroscopy.

### 2.6 Activation analysis using fNIRS

To investigate the activation in each brain region, the CBF change data for each participant were converted into a Z‐score for each measurement channel. The change in oxy‐Hb concentration derived by fNIRS was a relative value, and the values varied widely among the subjects because the optical path lengths differed depending on the subject’s cranial structure and the coordinates of the fNIRS probes. Therefore, the fNIRS data were normalized to the relative change in the baseline‐corrected data (Matsuda & Hiraki, 2006; Otsuka et al., 2007; Schroeter, Zysset, Kruggel, & Cramon, 2003; Shimada & Hiraki, 2006). The Z‐transformed data were baseline‐corrected so that the value at the beginning of the task block was zero, and the cumulative changes during the task block were then calculated. Moreover, because the task durations differed for the participants, the cumulative changes were divided by the number of samples during the task block of each participant. Because the task block was conducted three times, the cumulative changes for the three task blocks were averaged for each channel, and the mean value was utilized as the measure of brain activation.

### 2.7 Functional connectivity analysis

Functional connectivity is one of the most useful metrics for representing brain activity (Zhang et al., 2016). To investigate the functional connectivity network during the number memory task, Pearson correlations were calculated for the 70 channels of the oxy‐Hb time course during the task block in each auditory environmental condition. Finally, a 70 × 70 symmetric correlation matrix was obtained for each environmental condition. In addition, Fisher’s z‐transformation was performed so that the correlation coefficients were approximately normally distributed. A Fisher‐transformed correlation matrix is considered an adjacency matrix of a weighted undirected graph with the fNIRS measurement channel as the node and the correlation coefficient as the edge. Therefore, the matrix was analyzed using a graph theoretical analysis. The functional connection strength, which is one of the node centrality metrics in graph theory, was calculated for each correlation matrix to identify the important regions in the number memory task. The functional connection strength was derived as the sum of the weights of all adjacent links of a particular element (node) of the correlation matrix (Hampson, Driesen, Skudlarski, Gore, & Constable, 2006).

## 3 RESULTS

### 3.1 Comparison of the silent and white noise environments

The task performance, pleasantness rating, accumulated CBF change, and functional connection strength of all subjects were compared between the two experimental conditions, the silent and white noise environments, using *t* tests at a significance level of 5%. Performance and accumulated CBF change did not differ significantly. The pleasantness rating was significantly higher (better) in the silent environment than in the white noise environment [*t* (28) = 5.08, *p* < 0.05]. The functional connection strengths of the measurement channels associated with the premotor and supplementary motor cortices and visual association cortex were significantly higher in the silent environment than in the white noise environment [CH 18: *t* (28) = 2.56, *p* < 0.05; CH 67: *t* (28) = 2.09, *p* < 0.05]. Although the pleasantness level was significantly higher in the silent environment than in the white noise one, task performance and accumulated CBF change did not differ significantly. These results suggested that the auditory noise affected the functional connectivity of the premotor and supplementary motor cortices and visual association cortex. However, investigations of the effects of each experimental condition on intellectual work efficiency are difficult because the task performance did not vary between the conditions.

These results suggested that there were three different groups of the participants: (a) subjects whose task performances were improved in the noisy environment; (b) those not affected by the environmental noise; and (c) those whose task performances were affected negatively by the noise. Therefore, we divided the subjects into these three groups based on their task scores and analyzed the characteristics of each group.

### 3.2 Analysis of the behavioral data

**Figure 4:**
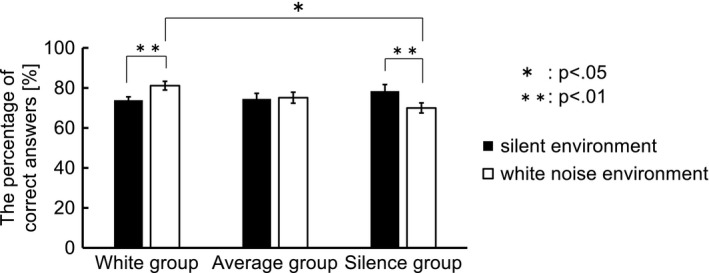
The task performances of the three groups. The subjects were divided into three groups based on their performance on the number memory task. The White, Average, and Silence groups contained 8, 13, and 8 subjects, respectively. The White and Silence groups differed significantly for task performance in the white noise and silent environments [White group: *t *(7) = 2.36, *p* < 0.01; Silence group: *t* (7) = 2.36, *p* < 0.01]. Moreover, the task performances of the White and Silence groups differed significantly in the white noise environment (*p* < 0.05 by Tukey’s multiple comparison test)

The subjects were divided into the three groups based on their performances on the number memory task. The difference in task performance (percentage of correct answers) between the silent and white noise environments was calculated. Subjects whose performance differences were higher than 3.2, which was half the standard deviation for all of the subjects, were called the White group, and those with performances within ±3.2 were called the Average group. The rest were called the Silence group. As a result, the White, Average, and Silence groups contained 8, 13, and 8 subjects, respectively. Figure 4 shows the task performances of the three groups. For the White and Silence groups, task performances in the white noise and silent environments differed significantly [White group: *t* (7) = 2.36, *p* < 0.01; Silence group: *t* (7) = 2.36, *p* < 0.01]. Moreover, the task performances of the White and Silence groups differed significantly in the white noise environment (*p* < 0.05 by Tukey’s multiple comparison test). These results (Figure 4) showed that the White group had better task performance in the white noise environment compared with that in the silent environment, and the Average group maintained its task performances in both environments. However, the Silence group had worse task performance in the white noise environment.

### 3.3 Subjective pleasantness

**Figure 5:**
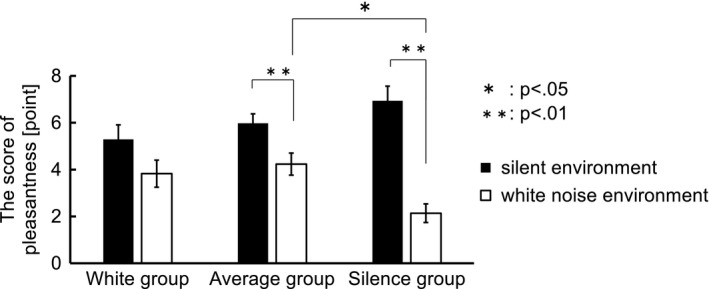
The pleasantness of the three groups. The Average and Silence groups differed significantly in the level of pleasantness in the silent and white noise environments [Average group: *t* (7) = 2.36, *p* < 0.01; Silence group: *t* (7) = 2.36, *p* < 0.01]. Moreover, the level of pleasantness in the white noise environment differed significantly between the Average and Silence groups (*p* < 0.05 by Tukey’s multiple comparison test)

Figure 5 shows the ratings for the level of pleasantness for the three groups. For the Average and Silence groups, the level of pleasantness differed significantly between the silent and white noise environments [Average group: *t* (7) = 2.36, *p* < 0.01; Silence group: *t* (7) = 2.36, *p* < 0.01]. Moreover, the level of pleasantness in the white noise environment differed significantly between the Average and Silence groups (*p* < 0.05 by Tukey’s multiple comparison test). The levels of pleasantness did not differ in the White group between the silent and white noise environments. The finding that some subjects in the White group who felt that the white noise environment was unpleasant were able to perform the task in the white noise environment after being in the silent environment was confirmed. The levels of pleasantness of the Average and Silence groups were lower in the white noise compared with the silent environment. The subjects in the Silence group felt that the white noise environment was more unpleasant than the Average group did.

### 3.4 Task activation analysis

**Figure 6:**
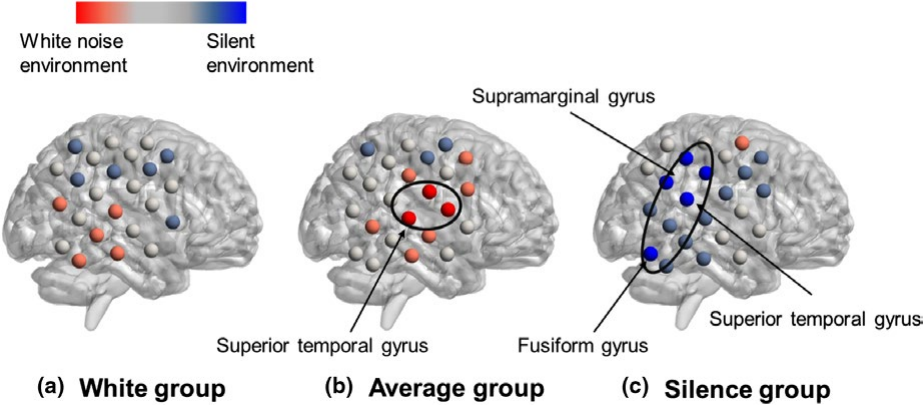
The cerebral blood flow (CBF) changes in the right temporal area. The color bars indicate the mean difference in task activation. The blue‐colored regions exhibited greater activation in the silent than in the white noise environments, whereas the red regions exhibited greater activation in the white noise environments. Here, *t* tests at a significance level of 5% were used to show differences in the accumulated CBF changes in each channel in the silent and white noise environments for each group. (a) The White group had no channels (regions) with significant differences between the two environments. (b) The Average group differed significantly (at a significance level of 5%) between the two environments for the channels associated with the right superior temporal gyrus. In these channels, the accumulated CBF changes were larger in the white noise than in the silent environments. (c) The accumulated CBF changes differed significantly (at a significance level of 5%) between the environments for the channels associated with the right fusiform gyrus, and they exhibited larger values in the silent environments. The accumulated CBF changes in the channels associated with the superior temporal gyrus and the supramarginal gyrus indicated larger values in the silent environments

**Figure 7:**
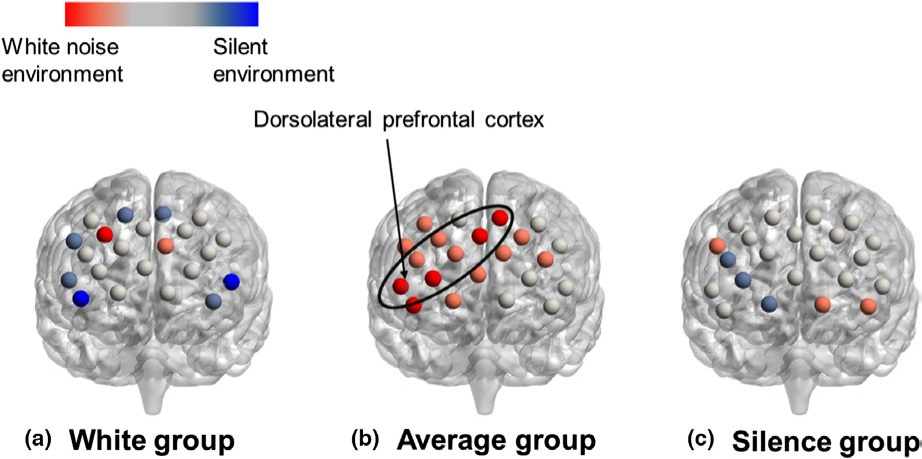
The cerebral blood flow (CBF) changes of the left temporal area. For the Average group, there was a significant difference (at a significance level of 5%) between the two environments for the channels associated with frontal pole, dorsolateral prefrontal cortex, and the inferior frontal gyrus. In these channels, the accumulated CBF changes were larger in white noise than silent environments

**Figure 8:**
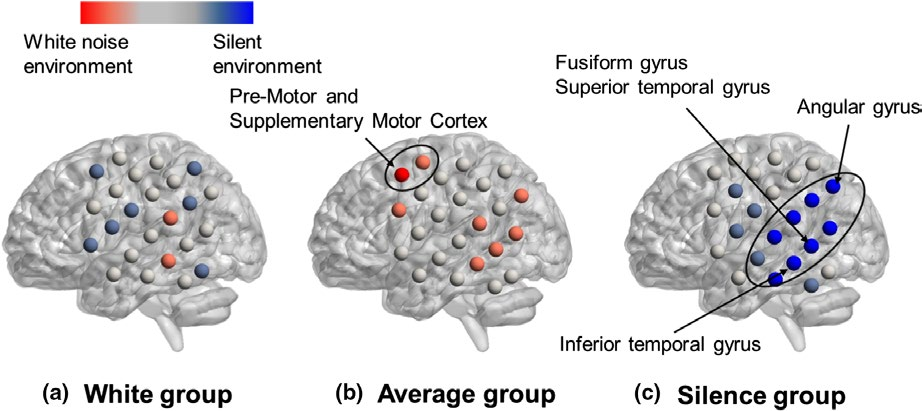
The cerebral blood flow (CBF) changes in the frontal area. The accumulated CBF changes in the channels associated with the superior temporal gyrus, inferior temporal gyrus, fusiform gyrus, and angular gyrus indicated larger values in silent environments

To better understand the difference in the task‐related activation between the silent and white noise environments, the accumulated CBF change in the silent environment was subtracted from that in the white noise environment, and the difference was meaned for each group. Figure 6 shows the mean differences in the task‐related activation in the right temporal regions in the three groups. Figure 7 shows the mean differences in the task‐related activation in the left temporal regions, and Figure 8 shows the mean differences in the task‐related activation in the frontal regions. The colored bars in the figures indicate the mean difference in task activation. The blue regions exhibited greater activation in the silent environment than in the white noise environment, whereas the red regions exhibited greater activation in the white noise environments. Furthermore, *t* tests were used at a significance level of 5% to test for differences in the accumulated CBF change of each channel in the silent and white noise environments for each group (Table 4). The results indicated that, for the White group, no channels (regions) had significant differences between the two environments. For the Average group, the two environments differed significantly (at a significance level of 5%) in the channels associated with the right superior temporal gyrus, subcentral area, frontal pole, dorsolateral prefrontal cortex, and inferior frontal gyrus. Moreover, in these channels, the accumulated CBF changes were larger in the white noise than in the silent environments. In contrast, the accumulated CBF changes in the Silence group differed significantly (at a significance level of 5%) between the environments in the channels associated with the right fusiform gyrus, which exhibited larger values in the silent environments. Furthermore, as shown in Figure 6, the accumulated CBF changes in the channels associated with the superior temporal gyrus, middle temporal gyrus, inferior temporal gyrus, fusiform gyrus, angular gyrus, and supramarginal gyrus were larger in the silent environment.

**Table 4:** *p*‐Value of the accumulated CBF change of each channel in the silent and white noise environments for each group

CH number	White group	Average group	Silence group
1	0.709	0.734	0.751
2	0.287	0.313	0.418
3	0.101	0.717	0.453
4	0.474	0.457	0.775
5	0.903	0.209	0.579
6	0.086	0.419	0.589
7	0.763	0.585	0.035
8	0.576	0.058	0.680
9	0.418	0.016	0.414
10	0.739	0.242	0.151
11	0.633	0.140	0.152
12	0.672	0.015	0.141
13	0.986	0.509	0.148
14	0.274	0.317	0.154
15	0.400	0.660	0.293
16	0.171	0.167	0.299
17	0.207	0.896	0.205
18	0.251	0.411	0.333
19	0.841	0.088	0.690
20	0.878	0.569	0.069
21	0.418	0.516	0.825
22	0.732	0.168	0.298
23	0.568	0.934	0.591
24	0.291	0.306	0.717
25	0.918	0.518	0.437
26	0.617	0.771	0.170
27	0.440	0.571	0.678
28	0.412	0.482	0.582
29	0.259	0.244	0.056
30	0.431	0.776	0.418
31	0.088	0.794	0.632
32	0.575	0.227	0.299
33	0.992	0.615	0.174
34	0.248	0.744	0.419
35	0.867	0.219	0.088
36	0.197	0.287	0.123
37	0.305	0.643	0.327
38	0.932	0.185	0.615
39	0.244	0.386	0.182
40	0.868	0.772	0.651
41	0.736	0.989	0.372
42	0.571	0.221	0.284
43	0.738	0.286	0.731
44	0.889	0.348	0.620
45	0.359	0.015	0.495
46	0.466	0.281	0.918
47	0.697	0.568	0.621
48	0.812	0.278	0.841
49	0.419	0.933	0.594
50	0.751	0.475	0.655
51	0.689	0.270	0.721
52	0.195	0.059	0.676
53	0.115	0.913	0.662
54	0.766	0.500	0.887
55	0.667	0.106	0.717
56	0.474	0.023	0.465
57	0.138	0.013	0.749
58	0.487	0.276	0.970
59	0.290	0.130	0.963
60	0.662	0.278	0.809
61	0.684	0.194	0.463
62	0.965	0.874	0.843
63	1.000	0.299	0.747
64	0.621	0.070	0.526
65	0.244	0.389	0.853
66	0.300	0.290	0.703
67	0.881	0.851	0.528
68	0.174	0.047	0.642
69	0.252	0.633	0.777
70	0.607	0.327	0.682

Note

CBF: cerebral blood flow.

### 3.5 Functional connectivity analysis

**Figure 9:**
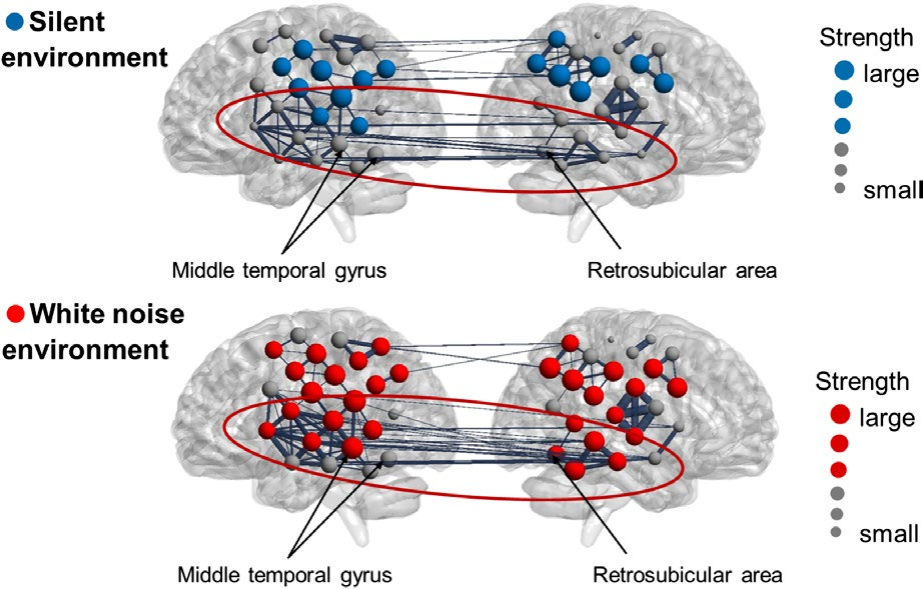
Functional connectivity network of the White group. The upper and lower panels indicate the functional networks in the silent and white environments, respectively. The nodes that had higher connection strengths than the mean of all subjects are colored, and the size of each node corresponds to the degree of strength. The connection strength of the White group was significantly larger (at a significance level of 5%) in white noise than in the silent environments in the middle temporal gyrus, right superior temporal gyrus, and left retrosubicular area. Moreover, the regions highly correlated with the above‐noted brain regions were the middle temporal gyrus, the superior temporal gyrus, the retrosubicular area, the subcentral area, the fusiform gyrus, the motor area, and the inferior frontal gyrus in both environments. The correlation coefficients among the neighboring regions and left–right correlations of the same region were higher in the white noise than in the silent environment

**Figure 10:**
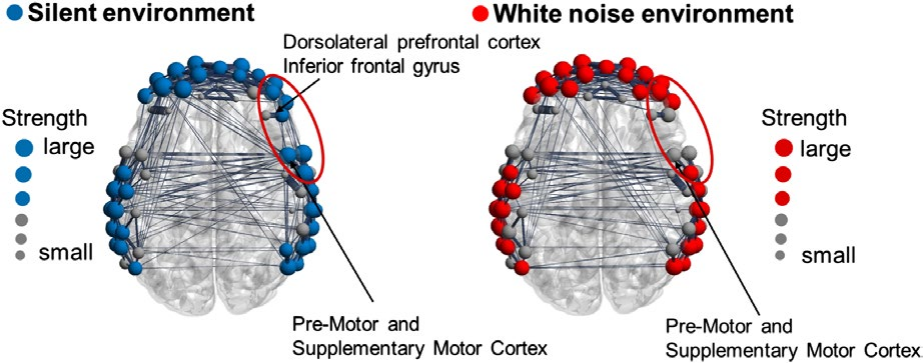
Functional connectivity network of the Average group. The connection strength was significantly larger (at a significance level of 5%) in the silent than in the white noise environments in the premotor cortex and supplementary motor area. Similarly, the premotor cortex and supplementary motor areas were highly correlated only with themselves and the subcentral area in white noise environment. In the silent environment, they were also highly correlated with the supramarginal gyrus, primary somatosensory cortex, superior temporal gyrus, dorsolateral prefrontal cortex, and inferior frontal gyrus

**Figure 11:**
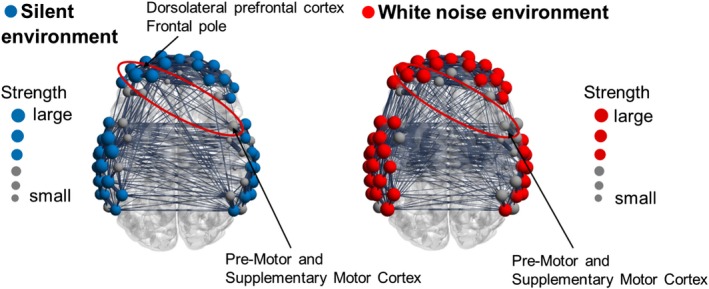
Functional connectivity network of the Silence group. The connection strength was significantly larger (at a significance level of 5%) in the silent than in the white noise environments in the premotor cortex and supplementary motor area. The correlation coefficients of the premotor cortex and supplementary motor area were higher only with themselves in the white noise environment, while they were also higher with the dorsolateral prefrontal cortex and frontal pole in the silent environment

The functional connection strength was calculated for each subject in the two environments to investigate the differences in the functional network structures among the three groups. Figure 9 shows the functional connectivity network of the White group, Figure 10 shows the network for the Average group, and Figure 11 shows the network for the Silence group. In each figure, the upper and lower panels indicate the functional networks in the silent and white environments, respectively. The nodes that had higher connection strengths than the mean of all subjects are colored, and the size of each node corresponds to the degree of strength. Table 5 shows the *p* value of the connection strength of each channel in the silent and white noise environments in each group. The connection strengths in the middle temporal gyrus, right superior temporal gyrus, and left retrosubicular area in the White group were significantly larger (at a significance level of 5%) in the white noise than in the silent environment. Moreover, the regions that were highly correlated with the brain regions listed above were the middle temporal gyrus, superior temporal gyrus, retrosubicular area, subcentral area, fusiform gyrus, motor area, and inferior frontal gyrus in both environments. Furthermore, the correlation coefficients among the neighboring regions and left–right correlations of the same region were higher in the white noise than in the silent environments. For the Average and Silence groups, the connection strengths in the premotor cortex and supplementary motor area were significantly larger (at a significance level of 5%) in the silent environment than in the white noise environment. Similarly, for the Average group, the premotor cortex and supplementary motor area were highly correlated only with themselves and the subcentral area in the white noise environment. However, in the silent environment, these areas were also highly correlated with the supramarginal gyrus, primary somatosensory cortex, superior temporal gyrus, dorsolateral prefrontal cortex, and inferior frontal gyrus. Finally, for the Silence group, the correlation coefficients of the premotor cortex and supplementary motor area were higher only with themselves in the white noise environment, and they were higher for the dorsolateral prefrontal cortex and frontal pole in the silent environment.

**Table 5:** *p*‐Value of the connection strength of each channel in the silent and white noise environments for each group

CH number	White group	Average group	Silence group
1	0.113	0.948	0.412
2	0.066	0.340	0.952
3	0.059	0.894	0.691
4	0.293	0.627	0.438
5	0.048	0.312	0.606
6	0.015	0.652	0.890
7	0.207	0.501	0.916
8	0.106	0.892	0.135
9	0.219	0.898	0.116
10	0.330	0.997	0.852
11	0.788	0.030	0.334
12	0.120	0.938	0.218
13	0.835	0.457	0.696
14	0.462	0.955	0.775
15	0.719	0.099	0.114
16	0.962	0.961	0.931
17	0.906	0.831	0.783
18	0.908	0.070	0.030
19	0.953	0.318	0.173
20	0.937	0.582	0.418
21	0.179	0.856	0.614
22	0.707	0.608	0.403
23	0.212	0.257	0.276
24	0.906	0.356	0.785
25	0.902	0.818	0.201
26	0.285	0.737	0.878
27	0.125	0.941	0.681
28	0.975	0.762	0.611
29	0.069	0.467	0.844
30	0.121	0.943	0.989
31	0.002	0.999	0.290
32	0.438	0.440	0.755
33	0.275	0.691	0.394
34	0.129	0.932	0.469
35	0.748	0.728	0.541
36	0.508	0.367	0.458
37	0.374	0.799	0.517
38	0.655	0.876	0.244
39	0.728	0.450	0.135
40	0.745	0.468	0.727
41	0.750	0.313	0.214
42	0.908	0.835	0.216
43	0.430	0.527	0.994
44	0.661	0.657	0.246
45	0.726	0.144	0.087
46	0.439	0.898	0.920
47	0.300	0.498	0.389
48	0.590	0.161	0.899
49	0.523	0.894	0.108
50	0.387	0.959	0.829
51	0.333	0.577	0.647
52	0.600	0.857	0.304
53	0.570	0.747	0.103
54	0.641	0.846	0.154
55	0.243	0.913	0.755
56	0.870	0.295	0.561
57	0.705	0.914	0.480
58	0.395	0.620	0.970
59	0.249	0.817	0.610
60	0.637	0.723	0.605
61	0.563	0.580	0.552
62	0.276	0.357	0.075
63	0.475	0.425	0.406
64	0.842	0.676	0.262
65	0.771	0.695	0.899
66	0.476	0.791	0.852
67	0.273	0.937	0.069
68	0.712	0.505	0.719
69	0.499	0.707	0.604
70	0.247	0.815	0.620

## 4 DISCUSSION

### 4.1 Analysis of the behavioral data and subjective pleasantness

The behavioral data analysis indicated there were three types of subjects: (a) subjects whose task performances were higher in the white noise than in the silent environments; (b) subjects whose task performances were not affected by the auditory environments; and (c) subjects whose task performances deteriorated in the white noise environments. We assumed that the psychological states of the three groups in the auditory environments differed from each other. With this assumption, we concluded that, because the White group was pleased by the white noise, their performance improved in that environments, while the performance of the Silence group, who were not pleased by the white noise, declined. The Average group who were not as unhappy with the white noise as the Silence group was were able to sustain their performance. These observations suggested that auditory environments that negatively affect the subjects also negatively affect their task performances. Thus, the task performances were related to the subjects’ psychological states.

### 4.2 Task activation analysis

Because the task performance of the White group differed between the white noise and silent environments, we expected that the CBF changes would differ according to the auditory environment. However, no significant differences were found. The results for the White group suggested that two types of subjects were intermixed: those whose performances were improved by their feeling comfortable with the white noise and the others whose performances were improved by getting used to the experimental environment, despite their feeling unpleasant in the white noise environment. We speculated that this was because there were no group tendencies in the CBF changes. However, the right superior temporal gyri of the Average group were more activated in the white noise than in the silent environment. The superior temporal gyrus contains the auditory cortex that is involved in auditory processing (Bigler et al., 2007). Moreover, this area is functionally responsible for phonological retention (Buchsbaum, Hickok, & Humphries, 2001). Because the right part of this region is involved in nonverbal auditory processing, it is probably activated when the subject is listening to white noise. The frontal pole of the forehead is responsible for future predictions, while the dorsolateral prefrontal cortex and inferior frontal gyrus play important roles in attention control and working memory tasks (Curtis & D'Esposito, 2003; Kane & Engle, 2002; Okuda et al., 2003; Rypma, Prabhakaran, Desmond, Glover, & Gabrieli, 1999). Activation of these three regions in the number memory task suggested that the subjects predicted the occurrence of unpleasant white noise and then tried to sustain their attention to the task. This might explain why the White group was able to maintain their task performance, even in the white noise environment, which they considered unpleasant. In the Silence group, the right and left fusiform gyri and its neighboring regions were more activated in the silent environment than in the white noise environment. The fusiform gyrus is responsible for recognizing various patterns, such as faces, words, and numbers (McCandliss, Cohen, & Dehaene, 2003). Furthermore, a wide area of the temporal region is activated during working memory tasks or tasks related to visual perception (Herath et al., 2001; Ishai, Ungerleider, Martin, Schouten, & Haxby, 1999; Onitsuka et al., 2004; Strand, Forssberg, Klingberg, & Norrelgen, 2008). These results suggested that the memorizing and recognizing of the visual numbers in the number memory task activated these regions. Moreover, the CBF changes in the Silence group were larger in the silent environment in which the subjects achieved better task performances and were decreased in the white noise environment in which the performances were worse. This finding suggested that the task‐related regions in the Silence group who were not pleased with the white noise differed markedly between the environments.

### 4.3 Functional connectivity analysis

In contrast, the difference in the CBF changes between the environments was not observed in the White group. However, the structure of the functional brain network differed. Because the connection strength around the middle temporal gyrus was larger in the white noise than in the silent environment, we inferred that the task performances were improved by this area working cooperatively with other adjacent brain regions. Similarly, the premotor cortex, supplementary motor area, and prefrontal cortex in the Average and Silence groups worked more cooperatively in the silent environment than in the white noise environment.

The premotor cortex and supplementary motor area were activated when the subjects entered answers by hitting the key during the number memory task. The supplementary motor area is especially important in planning sequential behavior based on the memory of order information (Tanji & Mushiake, 1996). In the silent environment, high functional connectivity among the motor cortex, supplementary motor area, and prefrontal cortex, which is related to attention control, is associated with the action of correctly and quickly entering the memorized numbers in order. In the Average group, the task performance did not differ between the environments, but the functional network differed. The functional connectivity between the supplementary motor area and prefrontal cortex was high in the silent environment. However, this connection was weakened in the white noise environment. We assumed that the effects of the white noise on the activity of the prefrontal cortex prevented its synchronization with the supplementary motor area. In the Silence group, the functional connectivity among the prefrontal cortex, premotor cortex, and supplementary motor area was lower in the white noise than in the silent environment. The deterioration of the task performance of the Silence group in the white noise environment might be associated with the decrease in these functional connections. Although this decrease was also observed in the Average group, their task performance did not deteriorate. Therefore, we assumed that the effects of white noise on attention control differed between the Average and Silence groups. The Average group might have been able to maintain its attention during the number memory task, even in the white noise environment, while the Silence group was distracted by the noise. These results suggested that the auditory environment that is appropriate for intellectual activities depends on the subject, and brain activity and functional brain networks can distinguish the different types of subjects.

### 4.4 Limitations of the study

The differences in the brain activity metrics of the accumulated CBF changes and the functional connection strengths depended on the subject type in this study. However, not all significant channels survived after the false discovery rate correction was applied (False discovery rate‐corrected *p *> 0.10). Multiple comparison corrections of the multichannel fNIRS measurements are a critical issue because it often results in a highly conservative analysis (Filippetti, Lloyd‐Fox, Longo, Farroni, & Johnson, 2015; Sato, Dresler, Haeussinger, Fallgatter, & Ehlis, 2014). A potential future direction is to develop a new correction method for the large number of channel settings and apply it to our research. Nonetheless, we believe that the findings of the current study were valuable.

## 5 CONCLUSION

In modern society, many people spend a considerable amount of time performing intellectual work, and auditory environments have been reported to affect their work efficiency. Therefore, investigations of auditory environments are needed to improve work efficiency. In this study, we investigated the relationship of brain activity and the performance of intellectual work based on the CBF change measurements performed using fNIRS and performance of the number memory task in noisy environments. Moreover, the psychological states of the subjects were estimated by subjective ratings of the pleasantness of the auditory environment. In the noisy environment, the participants performed the number memory task, while they listened to the auditory white noise. In addition, the experiment was conducted in an environment without white noise, and the results were compared. The subjects were divided into three groups based on their performances on the number memory task: The White group was positively affected by the white noise, the Average group was not affected, and the Silence group was negatively affected. The subjective ratings indicated that the White group was not affected by the white noise; hence, the CBF changes did not differ between the auditory environments. The left–right functional connections between the brain regions associated with the number memory task were strengthened, and task performance was improved. Although the Average group rated the white noise environment as unpleasant, the frontal regions related to attention control were more activated in the white noise environment, and this possibly related to attempts to maintain task performance, even in such noisy environments. In the Silence group, because the CBF changes in the regions related to the task in the Silence group were decreased in the white noise environment, the task performances might have been associated with the CBF changes. Moreover, the functional network analysis revealed that the premotor cortex and supplementary motor area worked cooperatively with the prefrontal cortex in the silent environment in which the task performance was higher. Our results suggested that the task performances were closely related to the level of pleasantness to the auditory white noise, and the effects of white noise on brain activity differed among the three groups based on the analysis of the CBF changes and functional connectivity networks. Furthermore, our results indicated that the optimal work environment in terms of auditory noise differed according to the subject. We categorized each subject into the three groups in this study using any of the three metrics: CBF change, pleasantness rating of the auditory noise, and score on the number memory task. The subject categorization will help to choose the auditory environment that is appropriate for each subject type. Therefore, we believe that our findings enabled us to quantitatively evaluate the effects of the auditory environment on intellectual work efficiency and contribute to the optimal design of individual work environments.
